# Structure-function analysis of Avian β-defensin-6 and β-defensin-12: role of charge and disulfide bridges

**DOI:** 10.1186/s12866-016-0828-y

**Published:** 2016-09-09

**Authors:** Ming Yang, Chunye Zhang, Xuehan Zhang, Michael Z. Zhang, George E. Rottinghaus, Shuping Zhang

**Affiliations:** 1 Department of Veterinary Pathobiology, University of Missouri, Columbia, MO USA; 2 Institute of Veterinary Medicine, Jiangsu Academy of Agricultural Sciences, Nanjing, People’s Republic of China; 3 Department of Biomedical Science, College of Veterinary Medicine, University of Missouri, Columbia, MO USA; 4 Department of Veterinary Pathobiology, Veterinary Medical Diagnostic Laboratory, College of Veterinary Medicine, University of Missouri, Columbia, MO 65211 USA

**Keywords:** Avian β-defensins, Antimicrobial activity, LPS-neutralizing activity, Chemotactic activity, Net positive charge, Disulfide bridges

## Abstract

**Background:**

Avian beta-defensins (AvBD) are small, cationic, antimicrobial peptides. The potential application of AvBDs as alternatives to antibiotics has been the subject of interest. However, the mechanisms of action remain to be fully understood. The present study characterized the structure-function relationship of AvBD-6 and AvBD-12, two peptides with different net positive charges, similar hydrophobicity and distinct tissue expression profiles.

**Results:**

AvBD-6 was more potent than AvBD-12 against *E. coli*, *S*. Typhimurium, and *S. aureus* as well as clinical isolates of extended spectrum beta lactamase (ESBL)-positive *E. coli* and *K. pneumoniae*. AvBD-6 was more effective than AvBD-12 in neutralizing LPS and interacting with bacterial genomic DNA. Increasing bacterial concentration from 10^5^ CFU/ml to 10^9^ CFU/ml abolished AvBDs’ antimicrobial activity. Increasing NaCl concentration significantly inhibited AvBDs’ antimicrobial activity, but not the LPS-neutralizing function. Both AvBDs were mildly chemotactic for chicken macrophages and strongly chemotactic for CHO-K1 cells expressing chicken chemokine receptor 2 (CCR2). AvBD-12 at higher concentrations also induced chemotactic migration of murine immature dendritic cells (DCs). Disruption of disulfide bridges abolished AvBDs’ chemotactic activity. Neither AvBDs was toxic to CHO-K1, macrophages, or DCs.

**Conclusions:**

AvBDs are potent antimicrobial peptides under low-salt conditions, effective LPS-neutralizing agents, and broad-spectrum chemoattractant peptides. Their antimicrobial activity is positively correlated with the peptides’ net positive charges, inversely correlated with NaCl concentration and bacterial concentration, and minimally dependent on intramolecular disulfide bridges. In contrast, their chemotactic property requires the presence of intramolecular disulfide bridges. Data from the present study provide a theoretical basis for the design of AvBD-based therapeutic and immunomodulatory agents.

## Background

Defensins are small cationic antimicrobial peptides containing six disulfide-paired cysteines [1]. Based on the sequence homology and connectivity of six conserved cysteine residues, defensins are classified into three subfamilies: α-, β- and θ-defensins [2–4]. Due to the interest in their potential application as antibiotic alternatives, defensins of different host species have been extensively investigated [5–8]. Although many defensins show broad-spectrum antimicrobial activities against bacteria, fungi, and some enveloped viruses, the mechanisms of action remain to be fully understood [8–11]. The antimicrobial mechanism of defensins primarily depends on several structural features, such as cationic charge and hydrophobicity, and is mainly divided into two classes, membrane-disruption and intracellular target [6, 12]. The membrane-disruptive model has been attributed to the electrostatic attraction between positively charged amino acid residues and negatively charged microbial membrane components (such as lipopolysaccharides (LPS), lipoteichoic acid (LTA) and anionic phospholipids) and insertion of hydrophobic residues into the microbial membrane, resulting in membrane disruption and cell death [10, 13]. Several models have been proposed to support this mechanism, including the barrel-stave, toroidal and carpet membrane pore-forming models, and sinking raft transient pore forming model [2, 14–18]. In addition to direct actions on microbial membrane, several studies have revealed intracellular functions of translocated antimicrobial peptides, such as interfering with cytoplasmic membrane septum formation and cell-wall synthesis, binding to nucleic acids, and inhibiting enzymes [2, 11, 15].

Defensins also contribute to adaptive immunity by chemoattracting monocytes, T lymphocytes, dendritic cells (DC) and mast cells to the site of infection, enhancing macrophage phagocytosis, inducing pro-inflammatory cytokines, and activating immature DCs [10, 11, 19]. The chemotactic activities of human β-defensin 1 to 3 (hBD1-3) as well as mouse β-defensin 2 (mBD2) are mediated by chemokine receptor CCR2 and CCR6 [20–23]. Activation of immature DCs by murine β-defensin-2 and avian β-defensin-13 involves the Toll-like receptor 4-nuclear factor-kappaB (TLR4-NFkB) signaling cascade [24, 25]. These activities enable β-defensins function as indigenous vaccine adjuvants [26, 27].

The chicken genome contains 14 β-defensin genes, located in a single defensin gene cluster on chromosome 3q3.5–3.7 [28, 29]. Some of these defensin peptides were initially referred to as gallinacins (Gal). To be consistent with the mammalian defensin nomenclature, the term of avian β-defensin (AvBD) was adopted [29–31]. The first two avian β-defensins, chicken AvBD-1 and AvBD-2, were isolated from chicken leukocytes in 1994, and later an inducible epithelially expressed avian β-defensin (AvBD-3) was reported in 2001 [32–34]. Transcriptional analysis of AvBD genes indicate that AvBD-1, −2, and −4 to −7 are mainly of myeloid origin, whereas the remaining AvBD-3, and −8 to −14 are mainly from epithelial cells. Both myeloid and epithelial AvBDs are expressed in a variety of other tissues [28, 29, 34]. Although many AvBDs possess certain degrees of antimicrobial activity and some may interact with immune cells, limited information is available regarding the mechanisms of antimicrobial and immunomodulatory activities [8, 11, 35]. In the present study, we have characterized the contribution of charge and disulfide bridges to various biological functions of AvBD-6 and AvBD-12 which are conserved in many avian species. While the hydrophobicity of these AvBDs are similar, AvBD-12 has the lowest average net positive charge and AvBD-6 has a relatively high net positive charge [36].

## Results

### Antimicrobial activity of AvBD-6 and AvBD-12

**Fig. 1:**
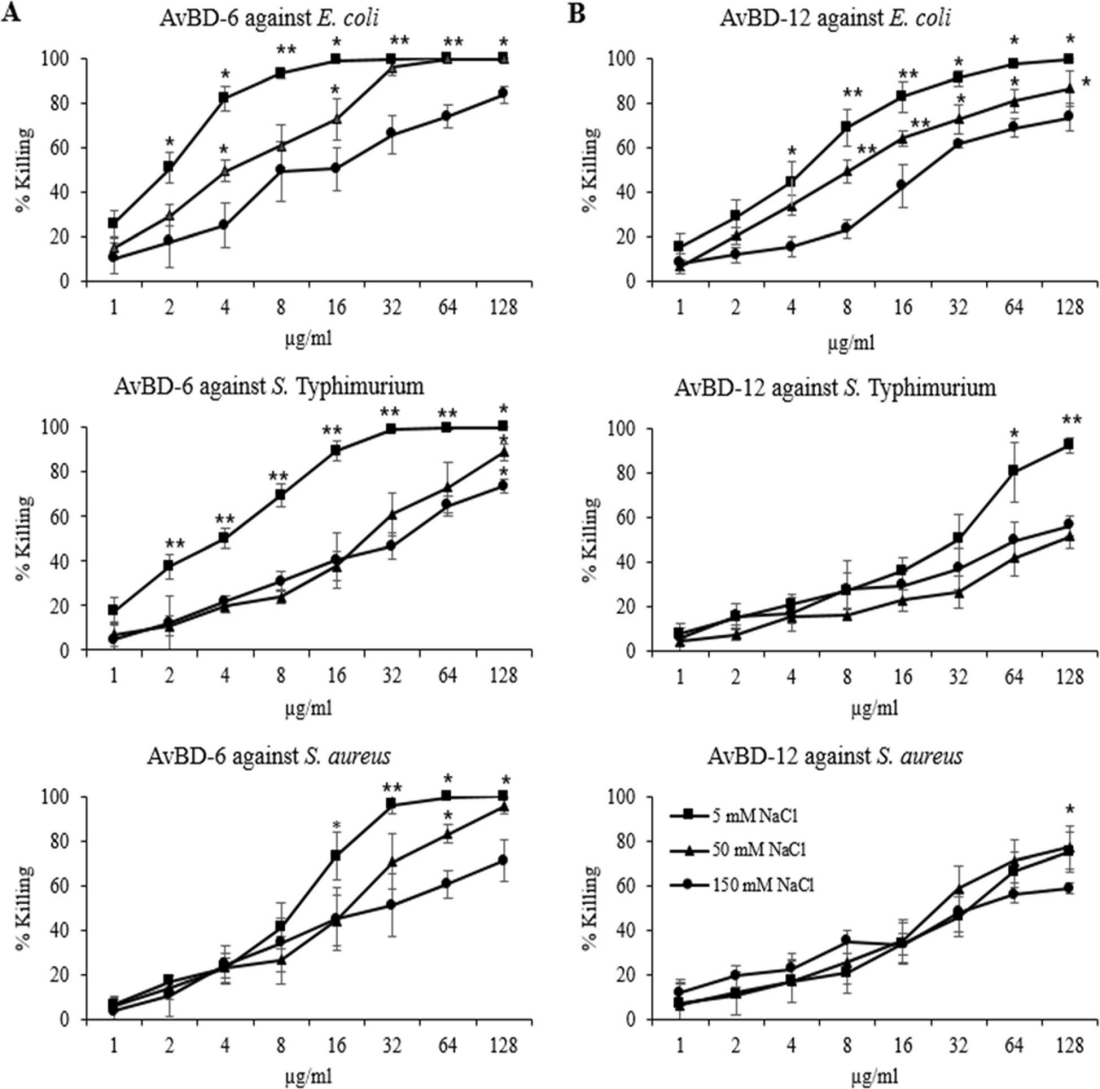
Antimicrobial activity of AvBD-6 and AvBD-12 against *E. coli*, *S.* Typhimurium and *S. aureus*. Bacteria (10^5^ CFU/ml) were incubated with various concentrations of AvBD-6 or AvBD-12 in the presence of 5 mM NaCl (■), 50 mM NaCl (▲), or 150 mM () at 37 °C for 3 h. **a** Antimicrobial activities of AvBD-6. **b** Antimicrobial activities of AvBD-12. Antimicrobial activity was presented as percent of killing as compared to no-AvBD control. Data are means of three independent experiments ± SD (*n* = 3). Asterisks indicate statistically significant difference between 5 mM and 50 mM and 150 mM of NaCl (*p < 0.05, **p < 0.01)

Both AvBDs showed dose-dependent (1 to 128 μg/ml) bactericidal activities against three common bacterial pathogens, *E. coli*, *S*. Typhimurium, and *S. aureus* (Fig. 1). To detect antimicrobial activity of AvBDs over a wide range of concentrations, percentage killing of bacteria was evaluated. AvBD-6 was generally more potent than AvBD-12 in killing *E. coli* (2 to 64 μg/ml), *S*. Typhimurium and *S. aureus* (8 to 128 μg/ml). The susceptibility of three bacterial pathogens to AvBDs (AvBD-6 at lower concentrations and AvBD-12 at higher concentrations) could be classified as: *E. coli* > *S*. Typhimurium > *S. aureus*. The killing activities of AvBDs were impaired by increasing NaCl concentration from 5 mM to 50 mM or 150 mM (Fig. 1). The negative impact of increased NaCl concentration could be summarized as follows: AvBD-6/AvBD-12/*E. coli* > AvBD-6/*S.* Typhimurium > AvBD-6/*S. aureus >* AvBD-12/*S*. Typhimuirum > AvBD-12/*S. aureus*.

**Fig. 2:**
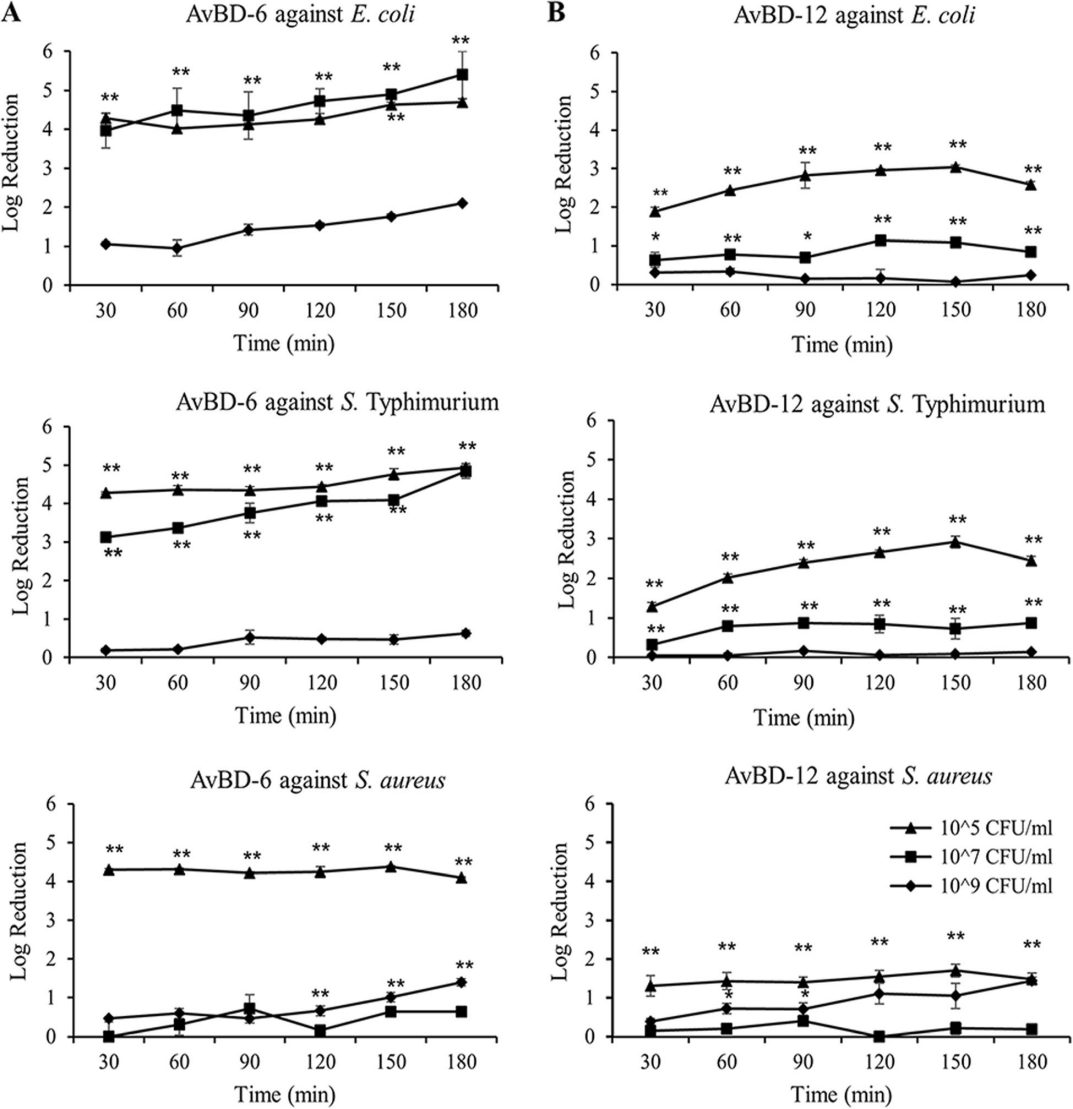
Killing kinetics of AvBD-6 and AvBD-12 against different bacterial concentrations. Bacteria (10^5^, 10^7^, and10^9^ CFU/ml) were treated with 32 μg/ml of at 37 °C for various times. **a** Killing kinetics of AvBD-6. **b** Killing kinetics of AvBD-12. Log reduction of CFU at each time point for each bacterial concentration was used to demonstrate the absolute number (relative to % killing) of bacteria by AvBDs. Data are presented as means ± SD (*n* = 3). Asterisks denote significant difference (*p < 0.05, **p < 0.01) in killing activity of each AvBD between different bacterial concentrations: 10^5^ CFU/ml (▲), 10^7^ CFU/ml (■) and 10^9^ CFU/ml (♦)

Since both AvBDs demonstrated significant killing activities at 32 μg/ml, this concentration was used to characterize killing kinetics. Log reduction of bacterial colony-forming unit (CFU) was used to define killing activity which enabled quantifying the number of bacteria killed when different concentrations of inoculum were tested. Kinetics study indicated that majority of the killing activity occurred within the first 30 min of bacteria-AvBD interaction (Fig. 2). Killing activity decreased significantly when bacterial inoculum concentration increased from 10^5^ CFU/ml to 10^9^ CFU/ml (*p* < 0.01). A comparison of the killing activities of both AvBDs confirmed that AvBD-6 was more potent than AvBD-12.

The minimum inhibitory concentrations (MICs) of both AvBDs against reference strains and clinical isolates were above 128 μg/ml (Table 2). When a low-salt Muller Hinton broth was used to determine MICs (MIC-ls), much lower values were obtained. For example, MIC-ls for AvBD-6 against ESBL-positive *E. coli* and *K. pneumoniae* were 8 μg/ml and 6 μg/ml, respectively.

### Ability of AvBD to neutralize LPS

**Fig. 3:**
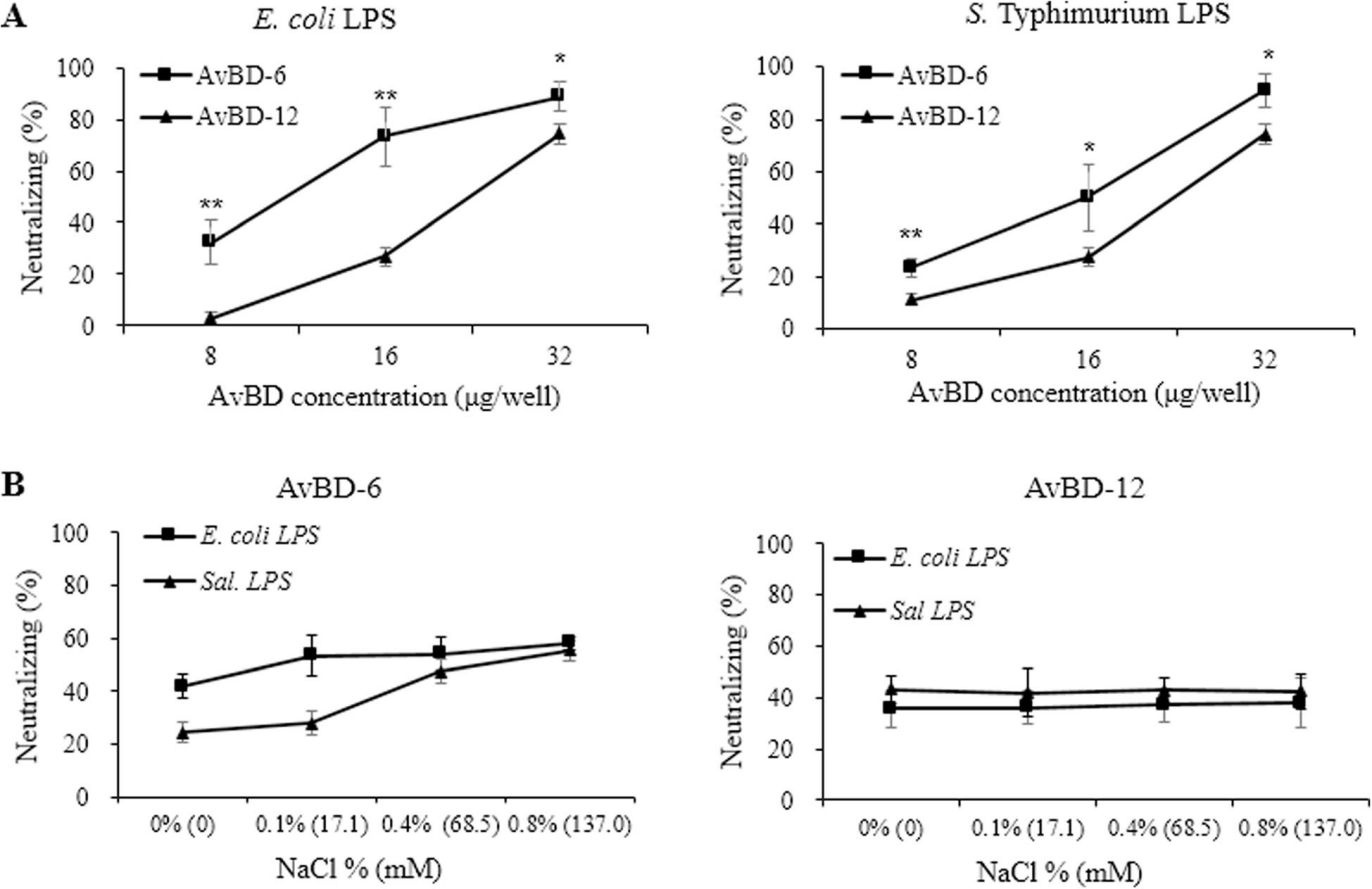
LPS-neutralizing activity of AvBD-6 and AvBD-12. LPS-neutralizing activities of AvBD-6 and AvBD-12 were determined by the Limulus Amoebocyte Lysate (LAL) assay. **a** Neutralizing activities AvBD-6 (■) and AvBD-12 (▲) for *E. coli* O111:B4 LPS and *S.* Typhimurium L6143 LPS. **b** The effect of NaCl concentration on the ability of AvBDs to neutralize *E. coli* O111:B4 LPS (■) and *S.* Typhimurium L6143 LPS (▲). The data are presented as means ± SD (*n* = 3). Asterisks denote statistically significant difference in LPS-neutralizing activities between AvBD-6 and AvBD-12 (*p < 0.05, **p < 0.01)

Both AvBD-6 and AvBD-12 neutralized LPS activity in a dose-dependent manner (Fig. 3a). At the concentration of 32 μg/ml, AvBDs were able to neutralize more than 70 % of equal volume of 1 EU/ml of LPS. The neutralizing capacity of AvBD-6 was significantly stronger (*p* < 0.05 or 0.01) than AvBD-12. While AvBD-6 (16 μg/ml) was more effective in neutralizing *E. coli* LPS than *Salmonella* LPS, AvBD-12 showed no difference in neutralizing *E. coli* LPS and *Salmonella* LPS. Interestingly, NaCl concentrations, ranging from 0.1 % (17.1 mM) to 0.8 % (137 mM), had no impact on AvBDs’ ability to neutralize LPS (Fig. 3b).

### Cell cytotoxicity

**Fig. 4:**
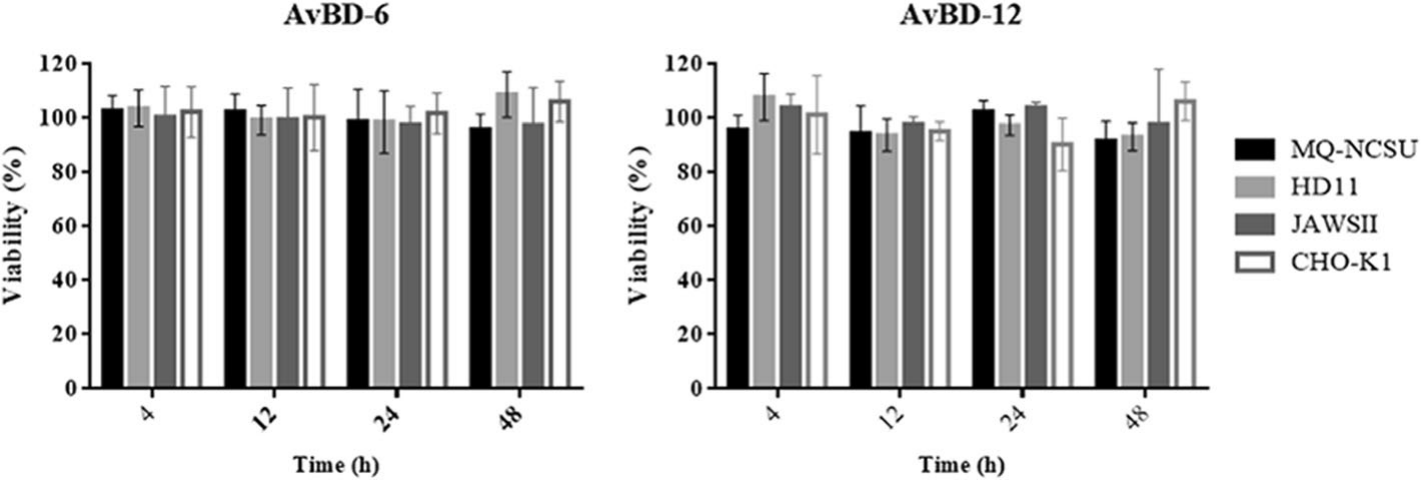
Cytotoxicity of AvBD-6 and AvBD-12 on host cells. Effect of 256 μg/ml AvBD-6 and AvBD-12 on the viability of chicken macrophages MQ-NCSU and HD11 cells, mouse immature dendritic JAWSII, and hamster CHO-K1 cells at 4, 12, 24, 48 h of incubation. Results shown are percentages of viable cells in different treatment groups relative to the untreated control cells. The data are expressed as the mean ± SD (*n* = 3)

The cellular toxicity of AvBD-6 and AvBD-12 to chicken macrophage cell line HD11 and MQ-NCSU, mouse immature dendritic JAWSII cells, and hamster CHO-K1 cells were evaluated using a MTT cell proliferation assay (Thermo Fisher Scientific). Exposure of cells to AvBDs at concentrations of 4, 16, 64, 256 μg/ml for 4, 12, 24, and 48 h did not cause any change in cell variability. Data on the highest concentration (256 μg/ml) and longest exposure (48 h) were presented in Fig. 4.

### Expression of CCR2-GFP/CCR6-GFP proteins in CHO-K1 cells

**Fig. 5:**
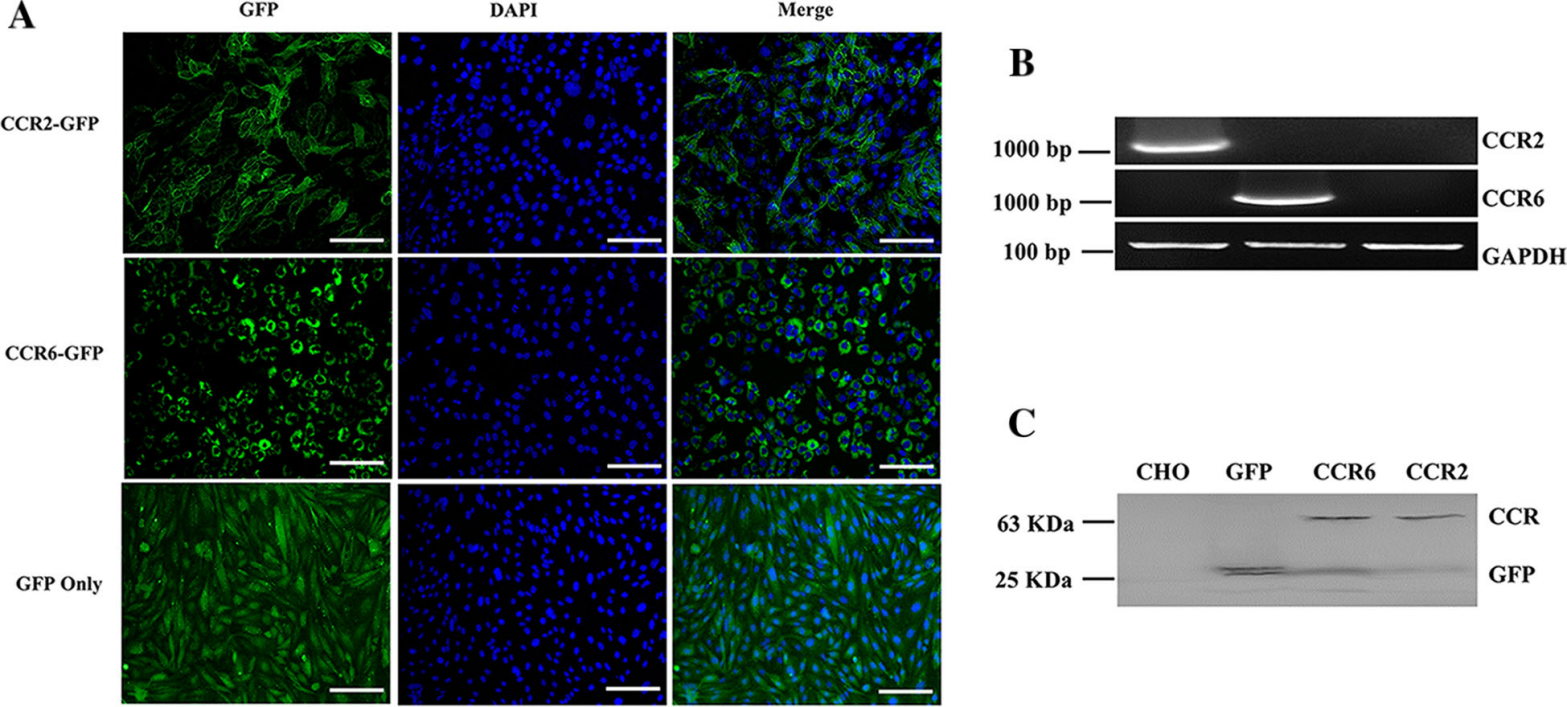
Expression of chicken chemokine receptors CCR2 and CCR6 in CHO-K1 cells. **a** Fluorescence images of CCR2-GFP, CCR6-GFP and GFP proteins (*green*) expressing CHO-K1 cells. Transfected cells were fixed with methanol: acetone (v:v, 1:1), and nuclei were stained with 0.1 μg/ml DAPI (*blue*). **b** Gel electrophoresis of RT-PCR products of CCR2 (1,065 bp) and CCR6 (1,089 bp) derived from transfected CHO-K1 cells. GAPDH (125 bp) was included as RT-PCR control. **c** Western-blotting analysis of the CCR2/CCR6-GFP fusion protein expressed in CHO-K1 cells. Wild-type and only GFP expressing CHO-K1 cells were included as negative and positive controls, respectively. The protein fractions were probed with polyclonal goat anti-GFP primary antibody (1:1000) and HRP-coupled anti-goat secondary antibody (1:500), and visualized after color development with 4-CN and 30 % hydrogen peroxide. GFP-CCR2 is primarily located in the cytoplasmic membrane, GFP-CCR6 in nuclear membrane, and GFP alone in cytoplasm. Scale bar: 100 μm

Fluorescent microscopy showed that CCR2-GFP fusion protein (green fluorescence) was mainly located in the cytoplasmic membrane of transfected CHO-K1 cells whereas CCR6-GFP was mostly found in the nuclear membrane and GFP alone was visible throughout the cytoplasm of CHO-K1 cells (Fig. 5a). Wild-type CHO-K1 cells did not show any green fluorescence. The expression of CCR2 and CCR6 in transfected cells was confirmed by RT-PCR which amplified the CCR2 and CCR6 genes with the expected sizes (1,065 bp for CCR2 and 1,089 bp for CCR6, shown in Fig. 5b). The expression of fusion proteins (CCR2-GFP, 65 kDa; CCR6-GFP, 66 kDa) was also confirmed by western blot analysis (Fig. 5c).

### Chemotactic activity of AvBDs

**Fig. 6:**
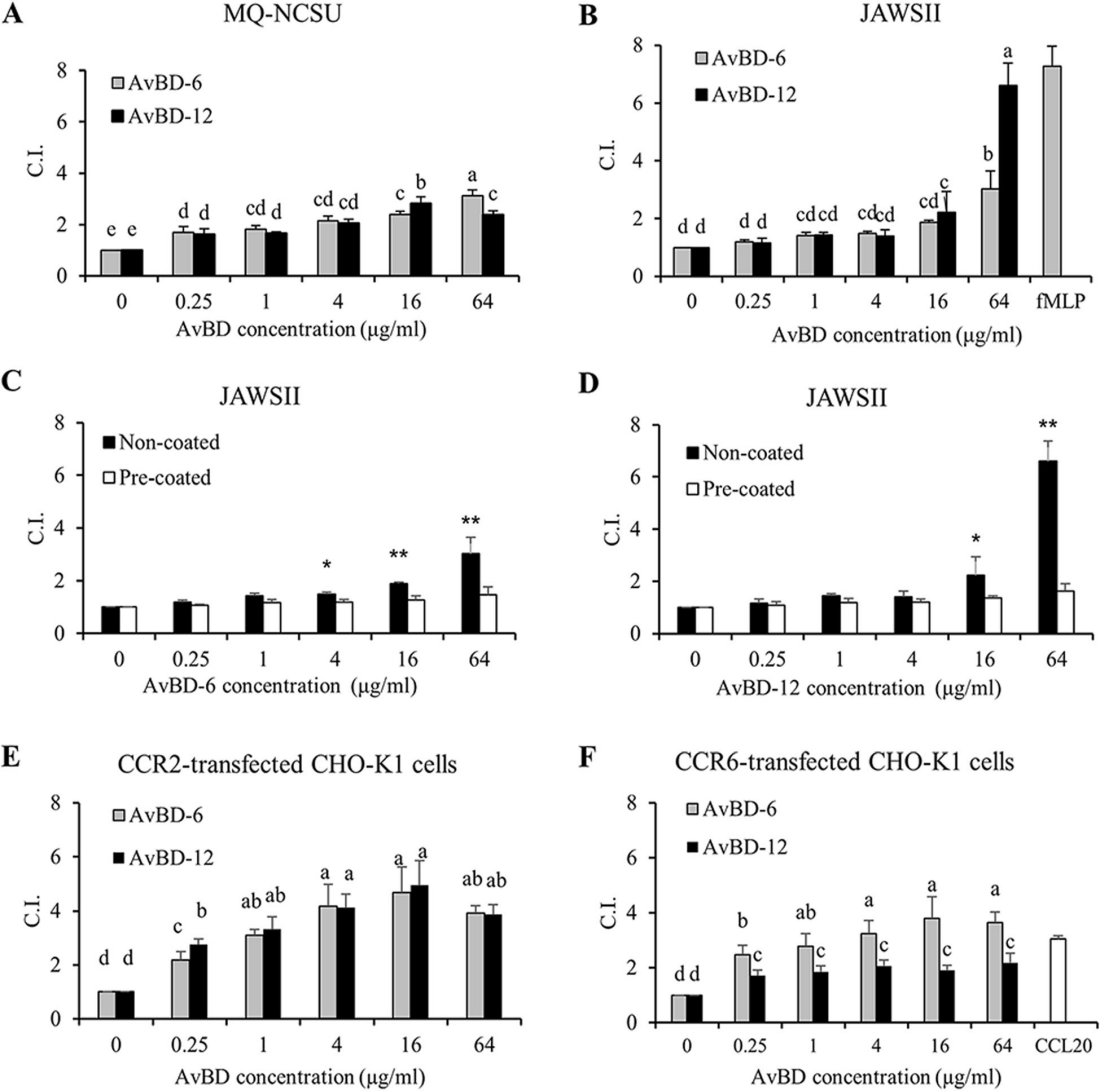
Chemotactic activity of AvBD-6 and AvBD-12. Migration of chicken macrophage MQ-NCSU cells (**a**), mouse immature dendritic JAWSII cells (**b**), AvBD-6 pretreated JAWSII cells (**c**), and AvBD-12 pretreated JAWSII cells (**d**), CCR2-positve CHO-K1 cells (**e**), CCR6-positive CHO-K1 cells (**f**). fLMP and CCL20 are used as positive control for dendritic cells and CCR6-transfected CHO-K1 cells, respectively. Chemotactic activities of AvBD-6 (□) and AvBD-12 (■) were expressed as chemotactic index (C.I.): = the number of migrated cells induced by AvBD/the number of migrated cells in response to chemotactic buffer. Data are presented as means of five independent experiments ± SD (*n* = 5). Different letters indicate significant difference (*p < 0.05). Asterisks indicate significant difference (*p < 0.05, **p < 0.01)

Both AvBDs demonstrated relatively low chemotactic activity for avian macrophage cell line MQ-NCSU (Fig. 6a). At a high concentration (64 μg/ml), both AvBDs induced the migration of murine immature dendritic cells (JAWSII) and AvBD-12 was significantly more effective than AvBD-6 in chemoattracting JAWSII cells (Fig. 6b). Pretreatment of JAWSII cells with AvBDs significantly inhibited subsequent cell migration towards AvBDs (*p* < 0.05), confirming that migration of JAWSII was induced by AvBDs (Fig. 6c and d). Both AvBDs exhibited a dose-dependent chemotactic effect on CCR2-CHO cells and no difference in their chemoattractant activities was detected (Fig. 6e). AvBD-6 also showed a dose-dependent chemotactic activity for CCR6-CHO cells whereas AvBD-12 demonstrated minimal chemotactic activity for CCR6-CHO (Fig. 6f).

### The antimicrobial, chemotactic, and neutralizing LPS activities of reduced AvBDs

**Fig. 7:**
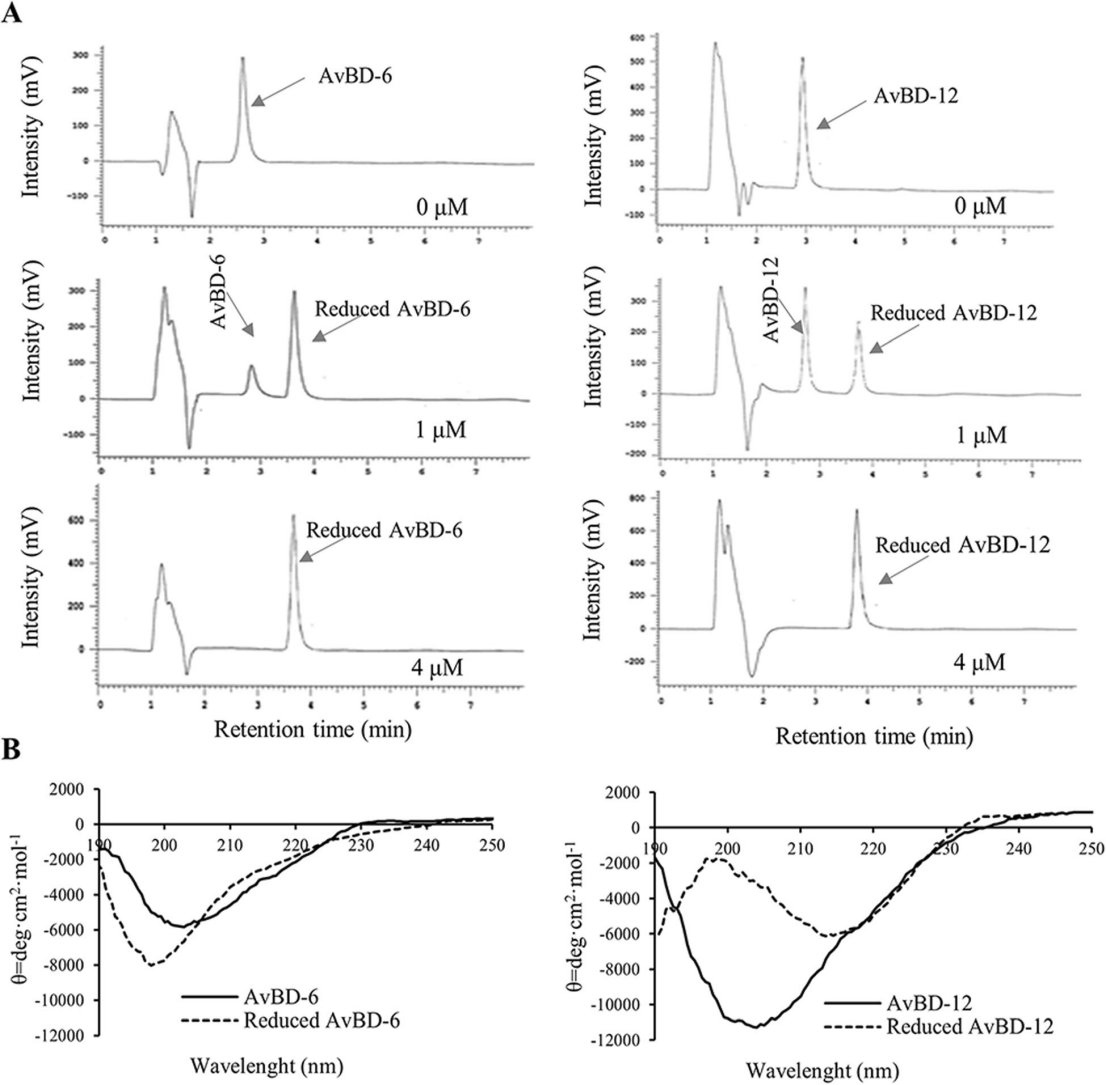
Reduced AvBD-6 and −12 were structurally different from wild-types AvBDs. **a** RP-HPLC analysis of AvBD-6 and AvBD-12 reduced by thioredoxin system with various concentrations of thioredoxin (0, 1, and 4 μM). Increased retention time and peak shifts indicate peptide unfolding and exposure of hydrophobic residues caused by thioredoxin treatment. Ten microgram of peptides was completely reduced by treatment with 4 μM thioredoxin. **b** Circular dichroism spectra of wild-type peptides (*solid lines*) and reduced peptides (*dotted lines*). Lines represent the average of six scans (*n* = 6). Wild-type peptides displayed intracellular β-sheet structure (signal at 205, 216 nm). In contrast, reduced peptides show a signal around 195 nm, indicated random coil structure. Molar ellipticity (θ) = deg.cm^2^.dmol^−1^

To evaluate the role of disulfide bridges in antimicrobial and chemotactic activities, AvBDs were reduced with a thioredoxin system (Fig. 7a). When defensins were fully reduced, the intramolecular disulfide bonds were broken and peptides became denatured which increased the overall hydrophobic surface area, compared to wild-type peptides with hydrophobic residues embedded inside. Reversed-phase high performance liquid chromatography (RP-HPLC) analysis of AvBDs following treatment with various concentrations of thioredoxin revealed that AvBDs were completely reduced by 4 μM thioredoxin as shown by the increased retention time with peak shifts (Fig. 7a). The structural changes due to removal of disulfide bonds were confirmed by far-ultraviolet circular dichroism (CD). Wild-type AvBD-6 and AvBD-12 displayed a broad negative band at 205 nm and a small negative shoulder at 216 nm, indicating a well-folded peptides with intracellular β-sheet conformation (Fig. 7b). In contrast, reduced AvBD-6 and AvBD-12 showed a significant negative signal around 195 nm, indicating a random coil structure. In addition, reduced AvBD-12 also displayed a negative shoulder at about 216 nm, which was likely caused by α-helix of AvBD-12. Data from RP-HPLC and CD analyses indicated that treatment with the thioredoxin system broke the intramolecular disulfide bonds.

**Fig. 8:**
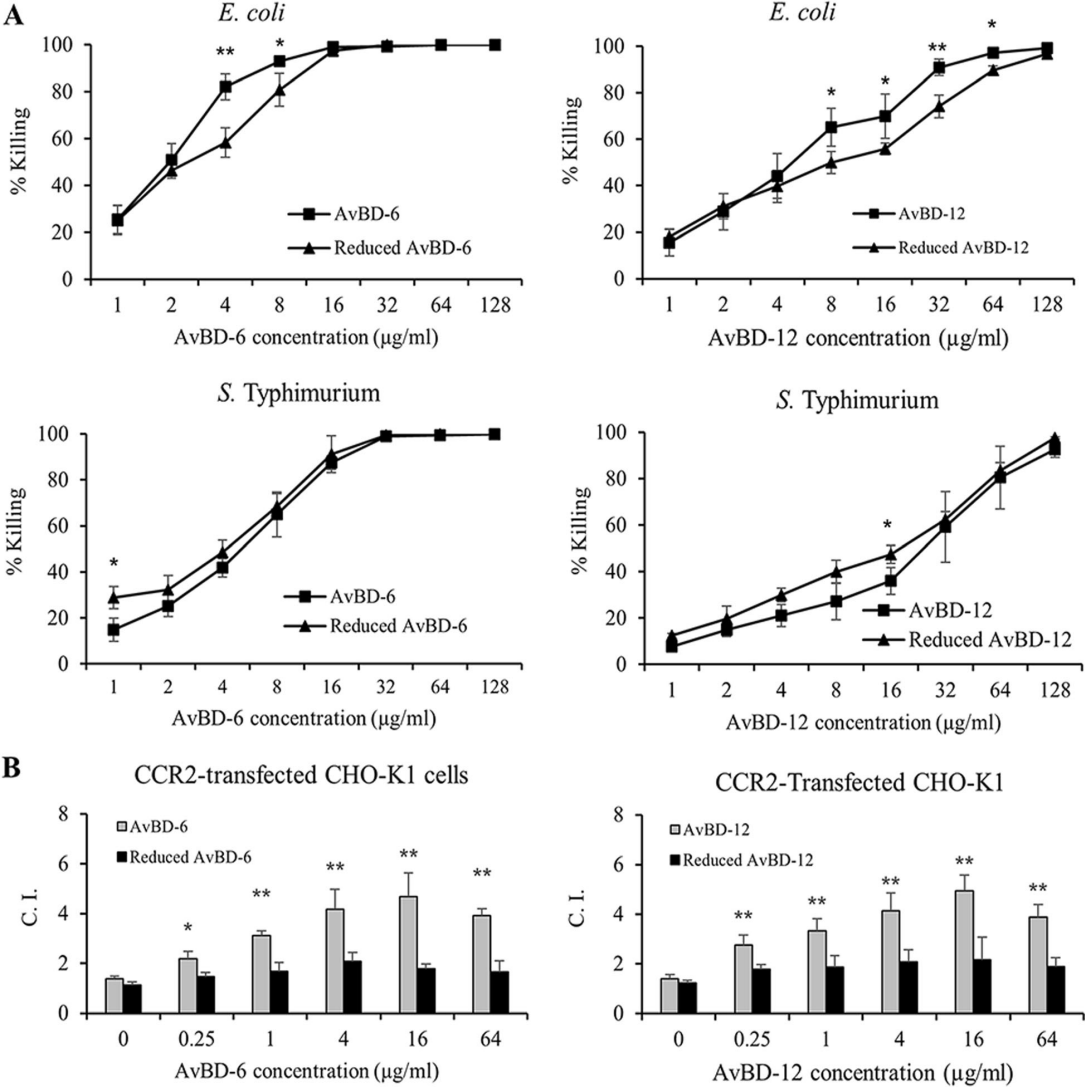
Antimicrobial and chemotactic activities of reduced AvBD-6 and AvBD-12. **a** Comparison of antimicrobial activity of reduced (■) and wild-type (▲) AvBDs against *E. coli* and *S.* Typhimurium. The assay was repeated three times and data are presented as means ± SD (*n* = 3). **b** Comparison of chemotactic activities of reduced and wild-type AvBDs. Chemotactic index (C.I.) = the number of migrated CCRs expressing CHO-K1 cells induced by AvBD/the number of migrated cells induced by chemotactic buffer. Data shown are means of three independent experiments ± SD (*n* = 5). Asterisks indicate significant difference of chemotactic activity between wild-type and reduced AvBDs (*p < 0.05, **p < 0.01)

**Fig. 9:**
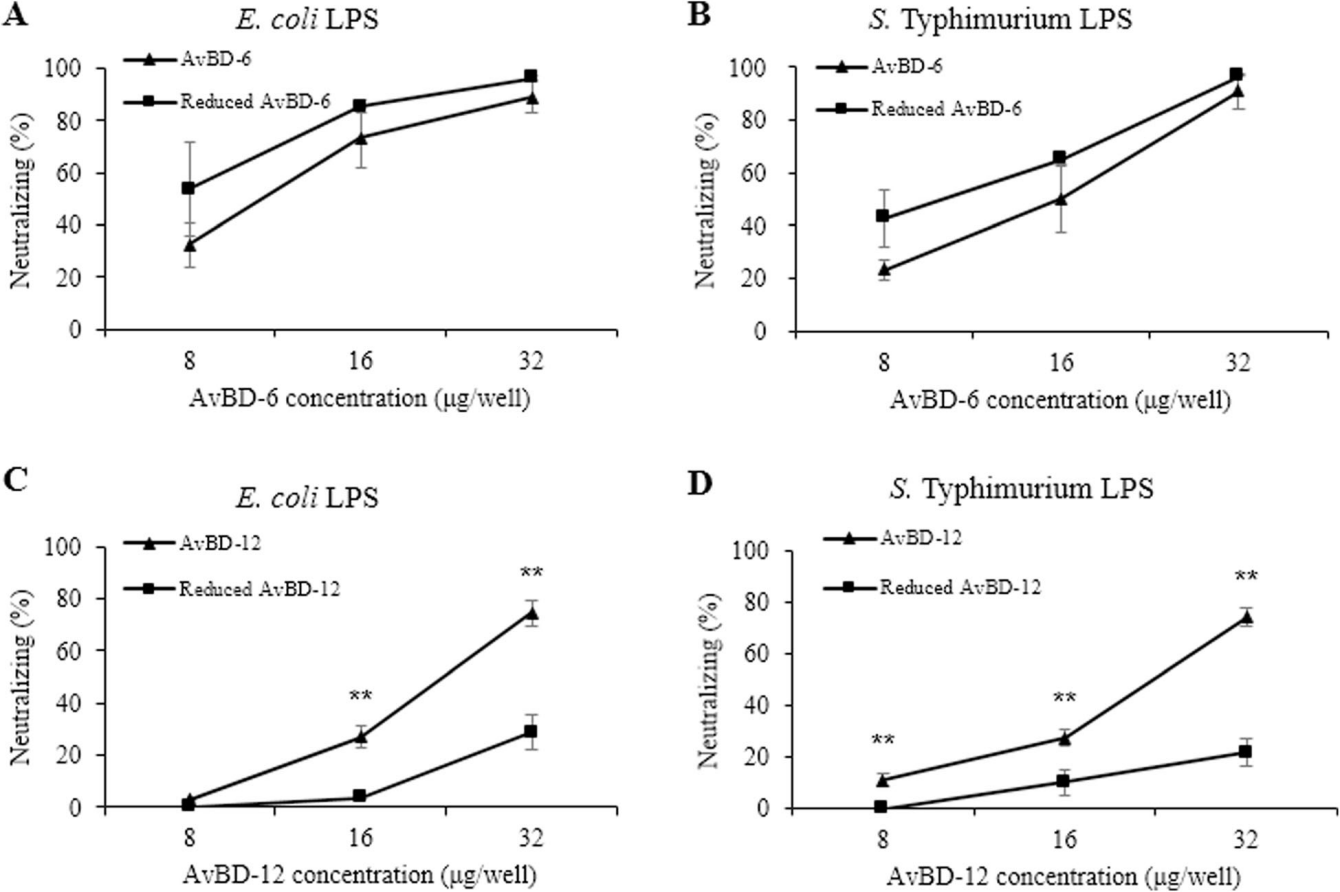
LPS-neutralizing activity of reduced AvBD-6 and AvBD-12. Comparison of LPS-neutralizing activity of reduced (■) and wild-type (▲) AvBDs. **a** Reduced AvBD-6 neutralizing for *E. coli* O111:B4 LPS, **b** Reduced AvBD-6 neutralizing S*.* Typhimurium L6143 LPS, **c** Reduced AvBD-12 neutralizing for *E. coli* O111:B4 LPS, **d** Reduced AvBD-12 neutralizing S*.* Typhimurium L6143 LPS. The assay was repeated three times and data are presented as means ± SD (*n* = 3). Asterisks indicate significant difference of neutralizing LPS activity between wild-type and reduced AvBD-12 (**p < 0.01)

The reduced AvBD-6 and AvBD-12 showed antimicrobial activities similar to that of the wild-type AvBD-6 and AvBD-12, respectively (Fig. 8a). In contrast, reduced AvBDs lost their chemotactic effect on CCR2-CHO cells (*p* < 0.01, Fig. 8b). Reduction had significant negative impact on the LPS-neutralizing activity of AvBD-12 (Fig. 9c and d). For example, wild type AvBD-12 at 32 μg/ml neutralized 74.48 % of *E. coli* LPS (1EU/ml) whereas reduced AvBD-12 neutralized 28.78 % *E. coli* LPS at the same peptide and LPS concentrations. Similar pattern was observed with *S.* Typhimurium LPS.

### TEM observations

**Fig. 10:**
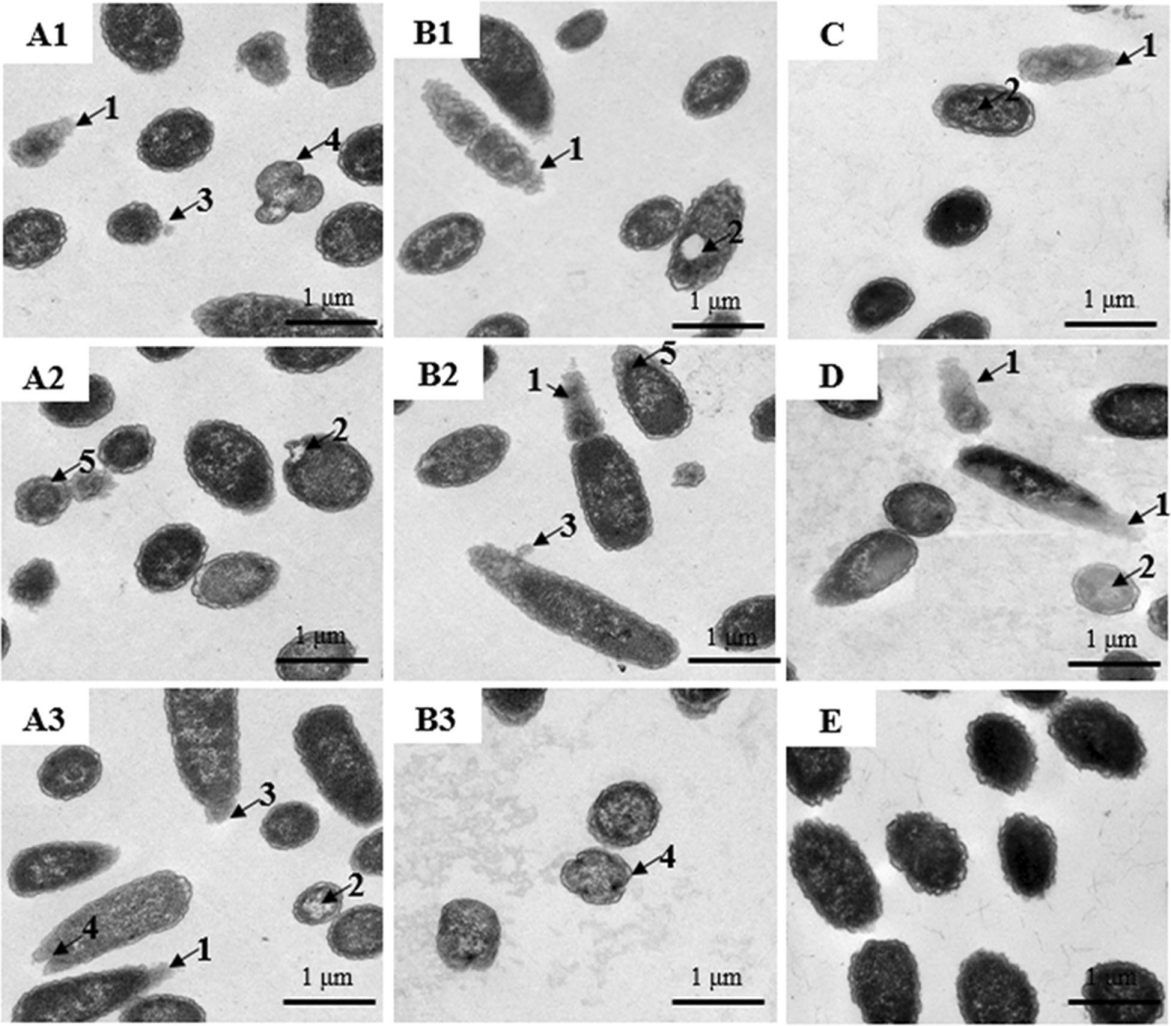
Transmission electron microscopy (TEM) analysis of AvBD-induced morphological changes of *S.* Typhimurium. Treatment of bacteria with wild-type and reduced AvBDs at 37 °C for 30 min resulted in fuzzy membrane (1), vacuole formation (2), membrane bleb (3), morphological change (4), and cytoplasm membrane shrinking (5). Bacteria were treated with AvBD-6 (**a**1-3), AvBD-12 (**b**1-3), reduced AvBD-6 (**c**), and reduced AvBD-12 (**d**). Untreated bacteria showed intact membrane, uniform cytoplasm (**e**). Figures are representatives of 10 images per treatment group. Approximately 29 % of bacteria treated with wild type AvBDs and 27 % of bacteria treated with reduced AvBDs displayed ultrastructural changes. Scale bar: 1 μm

Following treatment of S. Typhimurium with wild type and reduced AvBDs, ultrastructural changes were observed by TEM and the percentage of cells exhibiting ultrastructural changes were quantified based on 10 independent images per treatment group. Treatment of S. Typhimuriun with wild type AvBD-6 or AvBD-12 resulted in various ultrastructural changes, including fuzzy membrane, vacuole formation, membrane blebbing, and membrane shrinking (Fig. 10a and b). Treatment of bacteria with reduced AvBD-6 and AvBD-12 caused only fuzzy membrane and leakage (Fig. 10c and d). Approximately 29 % of bacterial cells treated with wild type AvBDs and 27 % bacterial cells treated with reduced AvBDs displayed ultrastructural changes. Untreated bacteria showed intact membrane, uniform cytoplasm without leakage of intracellular content (Fig. 10e).

### Binding of AvBDs to bacterial genomic DNA

**Fig. 11:**
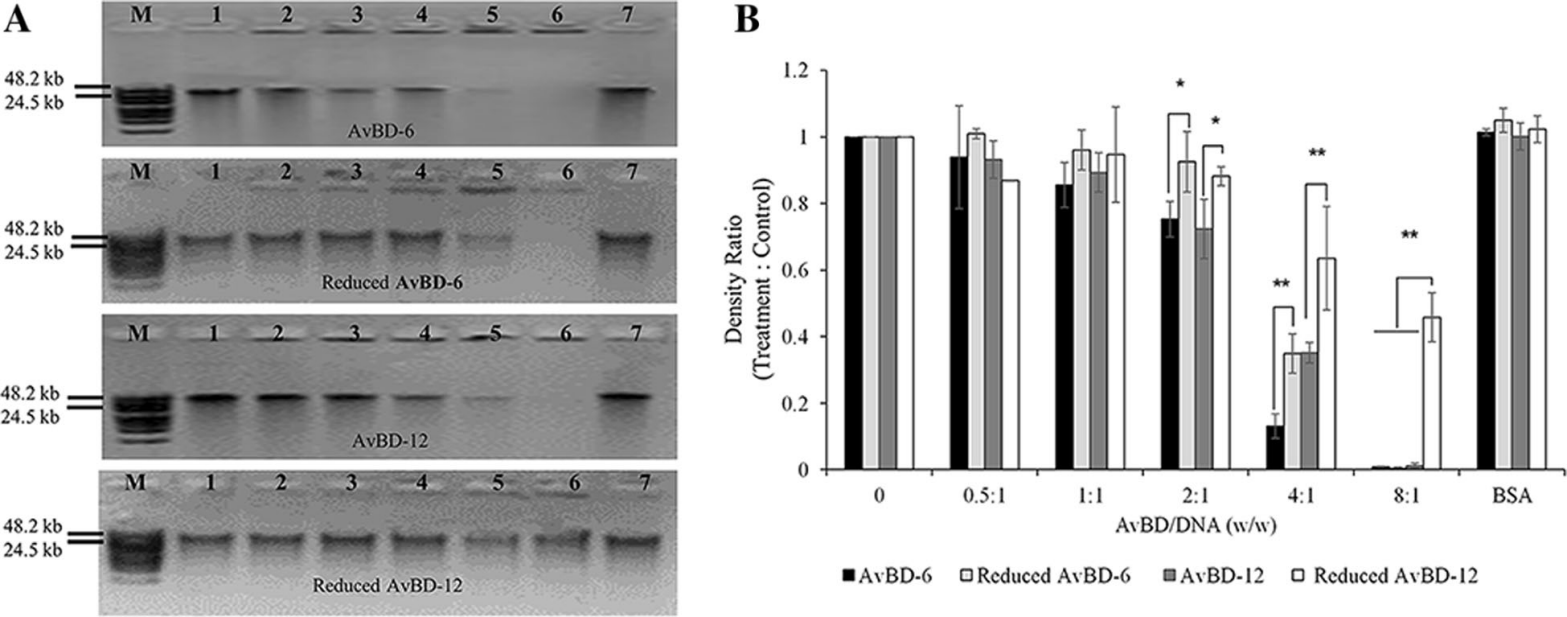
AvBD interaction with *S.* Typhimurium genomic DNA. **a** Gel retardation assay revealed the binding of wild-type and reduced AvBDs to *S.* Typhimurium genomic DNA. M: DNA molecular marker; lanes 1–6: the mass ratios of DNA to AvBD were 1:0, 1:0.5, 1:1, 1:2, 1:4 and 1:8, respectively; lane 7: DNA: bovine serum albumin (BSA) at ratio of 1:8. **b** Densitomeric analysis of migrated DNA by ImageJ software. Density ratio between treatment and control (lane 1) groups were calculated. Displayed values are means ± SD (*n* = 3). Asterisks indicate significant difference (*p < 0.05, **p < 0.01) between wild-type and reduced AvBDs

The ability of AvBDs to bind to bacterial genomic DNA was analyzed by a gel retardation assay [37]. Wild-type AvBDs at a mass ratio of 4:1 (AvBD:DNA) retarded more than 50 % of *S.* Typhimurium genomic DNA migration (Fig. 11). At a mass ration of 8:1 (AvBD:DNA), near complete retardation of genomic DNA migration by either AvBD was observed. BSA, as a negative control, had no effect at mass ratio of 8:1 (BSA:DNA). At a mass ratio of 4:1 (AvBD:DNA), AvBD-6 was significantly more effective than AvBD-12 in retarding genomic DNA migration (*p* < 0.01). Reduced AvBDs were less able than their respective wild-type peptides to retard *Salmonella* genomic DNA migration.

## Discussion

To understand the molecular mechanisms by which AvBDs contribute to host innate and acquired immunity, we have studied the transcriptional profiles of AvBD genes and characterized the antimicrobial activities of AvBDs with different net positive charges and tissue expression profiles [28, 29]. In the present study, we chemically synthesized the mature peptides of AvBD-6 and AvBD-12 and characterized the roles of peptide charge and intramolecular disulfide bridges in various biological functions. Our data indicated that synthetic AvBD-6 and AvBD-12 were highly effective in killing *E. coli*, *S*. Typhimurium, and *S. aureus* under low-salt condition. Compared to recombinant AvBDs, synthetic AvBDs demonstrated a potent antimicrobial activity against *S.* Typhimurium which was minimally susceptible to recombinant AvBDs in our previous study [38]. The discrepancy might be related to peptide folding because synthetic, but not recombinant AvBDs, were subjected to oxidative folding under optimal conditions. Consistent with our previous findings with recombinant AvBDs, the antimicrobial activity of AvBD-6 with a net positive charge of +7 was significantly higher than that of AvBD-12 with a net positive charge of +1. Since only two AvBDs were included in this study, it was difficult to truly assess the effect of charge on antimicrobial activity. Analysis of analog peptides is in progress to determine the contribution of charge and charge density to AvBDs’ antimicrobial activity. The antimicrobial potency of both AvBDs against *E. coli* and *S.* Typhimurium and AvBD-12 against *S. aureus* was negatively affected by increased salt concentration which was consistent with findings from previous investigations of cationic antimicrobial peptides [2, 15, 38, 39]. However, increased salt concentration had minor impact on the bactericidal activity of AvBD-12 against *S. aureus*, suggesting different mechanisms are utilized by different AvBDs to kill Gram-positive and Gram-negative bacteria. In this study, we also found that the antimicrobial effect of AvBDs was inversely associated with bacterial concentration, suggesting that a critical molecular ratio of AvBD:bacteria may be needed to cause microbial membrane damage. The antimicrobial activity of AvBDs (32 μg/ml) nearly diminished when bacterial concentration was increased from 10^5^ CFU/ml (CFU:AvBD molecule ratio = 1:4 × 10^2^) to 10^9^ CFU/ml (CFU:AvBD molecule ratio = 1:4 × 10^6^). The present study also evaluated the antimicrobial activities of AvBDs against clinical isolates of ESBL-positive *E. coli* and *K. pneumonia*e and methicillin resistant *S. pseudintermedius*. Although both AvBDs showed antimicrobial activity against ESBL-positive *E. coli* and *K. pneumoniae*, the MIC values ranged from 128 to 256 μg/ml, as compared to 4 to 64 μg/ml when the assays were conducted under low-salt condition. It has been reported that defensins interact electronically with bacterial membrane and thereby destroy the integrity of the membrane to kill the bacteria [10]. Therefore, it is important to determine whether these peptides can disrupt the host cellular membrane. In the present study AvBDs at a relatively high concentration (256 μg/ml) did not affect the viability of avian and mammalian cell lines examined. Our data indicate that AvBDs may be potential alternative therapeutic agent for infections caused by ESBL-producing bacteria if their sensitivity to salt can be modified.

It has been reported that the presence of disulfide bridges was not essential for bovine BNDB-2 and BNDB-12 or human hBD3 to exert their antimicrobial function [22, 40–43]. To investigate the structure-function relationship of AvBDs, we treated AvBDs with the thioredoxin reductase to break the disulfide bridges and confirmed the reduction of AvBDs by RP-HPLC and CD [44–46]. Reduced AvBDs showed slightly reduced potency against *E. coli* which was in line with a previous report that AvBD-6 without the three disulfide bonds expressed in *Pichia Pastoris* had antimicrobial activity against *S. aureus* and *Bacillus subtilis* [47].

As a major component of Gram-negative bacteria, LPS maintains the structural integrity of bacteria and causes septic shock or inflammatory responses in host [48]. It was previously shown that human defensin beta 123 (DEFB123) and recombinant human DEFB114 could inhibit LPS-mediated inflammation [49, 50]. It is known that electrostatic interactions between defensins and negatively charged LPS are prerequisite for the binding of peptides to Gram-negative bacteria and subsequent membrane damaging activities, and any factors (such as cationic salt concentration) interfering with the electrostatic attraction could inhibit defensins’ antimicrobial functions [51]. In the present investigation, AvBD-6 with a higher net positive charge was more able than AvBD-12 to neutralize LPS, but LPS-neutralizing capacity of neither AvBD was affected by increased NaCl concentration. In fact, both AvBDs were fully functional at near physiological concentration of NaCl (0.8 %, 137 mM). However, the LPS-neutralizing ability correlated with the net positive charges of AvBDs, suggesting that electrostatic attraction is an essential, but not the only factor that affects the interaction between AvBD and LPS. This was further supported by the fact that reduced AvBDs having the same net positive charges as their respective wildtype peptides were less able to neutralize LPS, specifically for AvBD-12.

Mammalian defensins can interact with cell membrane receptors to influence diverse cellular processes including antigen presentation, chemotaxis, and cytokine release [52, 53]. In the present investigation, both AvBDs demonstrated mild chemotactic activities for MQ-NCSU, and AVBD-12 at a relatively high concentration had strong chemoattractant effect on murine immature dendritic cells. Pretreatment JAWSII cells with AvBD blocked AvBD-induced cell migration (*p* < 0.05), suggesting that AvBDs interacted with murine dendritic cell membrane component(s), likely chemokine receptors which are highly conserved across species [54, 55]. We then determined if CCR2 and CCR6 are the receptors for AvBDs as indicated previously for hBD1-3 and mBD2 and mBD14 [20–23]. Both AvBD-6 and AvBD-12 displayed strong chemotactic effect on CCR2-positive CHO-K1 cells whereas only AvBD-6 induced moderate chemotaxis of CCR6-positive CHO-K1 cells. CCR6 as a G-protein-coupled receptor with seven transmembrane domains is expressed by memory T cells, B cells, and DCs and involved in recruiting leukocytes to sites of inflammation [56]. In the present study, fusion with GFP changed the location of CCR6, which might have affected the chemotactic migration of CCR6-positive CHO-K1 cells. We conducted several independent transfection assays with different clones of CCR6 plasmids, all of which resulted in the primary expression of CCR6-GFP in the nuclear membrane. Nonetheless, our data show that AvBDs are broad-spectrum chemoattractant molecules for avian and mammalian immune cells.

The dual antimicrobial mechanism involving targeting microbial membrane and nucleic acids and enzymes have been reported [57–62]. In the present study, we determined the role of disulfide bridges of AvBDs in damaging bacterial membrane and binding to genomic DNA. Treatment of *S*. Typhimurium with AvBDs resulted in fuzzy membrane, loss of cytoplasmic content, cytoplasmic membrane shrinkage, and morphological change (Fig. 10a and b) whereas reduced AvBDs caused only fuzzy membrane and leakage of intracellular content. Our data suggest that wild-type AvBDs with conserved disulfide bridges not only disrupted bacterial membrane but also interfered with cell division and other intracellular functions that cause morphological changes. In contrast, reduced AvBDs exert their bactericidal function mainly through membrane-lytic mechanism(s). Gel retardation assay ascertained that wild type AvBDs are more able than reduced AvBDs to bind bacterial genomic DNA, potentially interfering with transcription and translation of affected bacteria.

## Conclusions

AvBD-6 and AvBD-12 exhibited strong antimicrobial activities against *E. coli*, *S.* Typhimurium, and *S. aureus* under low-salt conditions. The antimicrobial activity was positively correlated with the peptides’ net positive charges, inversely correlated with bacterial concentration, strongly inhibited by NaCl at physiological concentration (150 mM), and minimally dependent on the intramolecular disulfide bridges. LPS-neutralizing activity of AvBDs was dependent on disulfide bridges (for AvBD-12) and unaffected by NaCl concentration. Chemotactic activity required the tertiary structure of AvBDs but not directly related to the peptide charge.

Functional characterization of two different AvBDs suggests that different mechanisms could be involved in their actions against different microbial pathogens and microbial products (such as LPS). Data from the present investigation provide the theoretical basis for future application of AvBDs or their analogues as therapeutic agents for bacterial infections and LPS-induced shock, as well as vaccine adjuvants for avian and mammalian species.

## Methods

### Peptides synthesis

The mature peptides of AvBD-6 (SPIHACRYQRGVCIPGPCRWPYYRVGSCGSGLKSCCVRNRWA [GenBank: AAT45546.1]) and AvBD-12 (GPDSCNHDRGLCRVGNCNPGEYLAKYCFEPVILCCKPLSPTPTK [GenBank: AAT48936.1]) were custom synthesized using the standard solid phase 9-fluorenylmethoxycarbonyl (Fmoc) method by LifeTein LLC (Hillsborough, NJ). Linear peptides were subjected to oxidative folding to ensure the correct formation of the three disulfide bridges between Cys^1^-Cys^5^, Cys^2^-Cys^4^ and Cys^3^-Cys^6^. Following confirmation by mass spectrometry, AvBDs were purified by reversed-phase high performance liquid chromatography (HPLC) and lyophilized (Lifetein, Hillsborough, NJ). The purity of the synthetic mature peptides was >98 %, the molecular weight of AvBD-6 and AvBD-12 were 4738.57 and 4892.76, and the net charge of AvBD-6 and AvBD-12 at pH 7.0 were +7 and +1, respectively, and they have same hydrophobicity (Table 1).

**Table 1:** Amino acid sequence and properties of AvBD-6 and AvBD-12

Peptide	Amino acid sequence^a^	Length (aa)	Molecular weight (Da)	Charge	Hydrophobicity
AvBD-6	SPIHAC^1^RYQRGVC^2^IPGPC^3^RWPYYRVGSC^4^GSGLKSC^5^C^6^VRNRWA	42	4738.57	+7	33 %
AvBD-12	GPDSC^1^NHDRGLC^2^RVGNC^3^NPGEYLAKYC^4^FEPVILC^5^C^6^KPLSPTPTKT	45	4892.76	+1	33 %

^a^The three disulfide bridges formed between Cys^1^-Cys^5^, Cys^2^-Cys^4^ and Cys^3^-Cys^6^

### Antimicrobial activity assay

*Escherichia coli* (*E. coli*, ATCC 25922), *Salmonella enteric* serovar Typhimurium (*S.* Typhimurium, ATCC 14028), and *Staphylococcus aureus* (*S. aureus*, ATCC 29213) were used to assess AvBDs’ antimicrobial activity. Extended spectrum beta lactamase (ESBL) positive clinical isolates of *E. coli*, *Klebsiella pneumoniae* (*K. pneumoniae*), and methicillin-resistant *S. pseudinetrmedius* (Table 2) were included in the antimicrobial assays. All bacterial strains were grown and maintained on Trypticase Soy Agar (TSA) with 5 % Sheep Blood (Thermo Fisher Scientific) at 37 °C.

**Table 2:** Minimum Inhibitory Concentrations of AvBD-6 and AvBD-12^a^

Microorganism	Source (Number of strains)	AvBD-6		AvBD-12	
		MIC	MIC-ls	MIC	MIC-ls
Gram-negative					
*E. coli*	ATCC 25922 (1)	128	4	256	32
*E. coli*	Clinical isolates (10)	256	≤8	256	≤64
*S. enterica* serovar Typhimurium	ATCC 14028 (1)	≥256	16	>256	128
*K. pneumoniae*	Clinical isolates (10)	≥256	≤16	>256	≤64
Gram-positive					
*S. aureus*	ATCC 29213 (1)	256	128	>256	256
*S. pseudinetrmedius*	Clinical isolates (10)	≥256	≥256	>256	≥256

^a^Minimum inhibitory concentrations (μg/ml) were determined using the CLSI broth microdilution method and the modified broth microdilution method using low salt Muller Hinton Broth (MIC-ls) as described in materials and methods section. Clinical isolates were received from the Clinical Veterinary Microbiology Laboratory of Texas A&M University

The bactericidal activity of AvBD-6 and AvBD-12 was determined by colony counting assay [38]. Three to five bacterial colonies from an overnight culture on a TSA agar plate were suspended in 5 ml sterile distilled water to achieve a MacFarland standard of 0.5 (~10^8^ colony forming units per milliliter, CFU/ml). Ten microliters of bacterial suspension was inoculated into 5 ml 100× diluted Mueller-Hinton broth (MHB) to obtain a final concentration of approximately 2 *×* 10^5^ CFU/ml. Twenty-five microliters of bacterial culture was mixed with 25 μl of 2-fold serially diluted AvBD-6 and AvBD-12 in a Nunc^™^ 96-well polypropylene microtiter plate (Thermo Fisher Scientific), and the final concentrations of AvBDs were 1, 2, 4, 8, 16, 32, 64, 128 μg/ml. Gentamycin (100 μg/ml) and peptide dilution buffer were used as positive and negative controls, respectively. Following an incubation at 37 °C for 3 h, 10-fold serial dilutions of the bacterial-peptide mixture were inoculated onto LB agar plates, then colonies were counted after 16 h of incubation at 37 °C. The bactericidal activity was expressed as percent of killing using the following formula: (CFU_control_ - CFU_treated_)/CFU_control_ × 100 %. To investigate the effect of ionic strength on the antimicrobial activity of AvBD-6 and AvBD-12, 5 mM, 50 mM, and 150 mM NaCl were included in the incubation buffer with peptides at antimicrobial assay.

MICs of AvBD-6 and AvBD-12 were determined using broth microdilution method according to the guidelines of Clinical and Laboratory Standards Institute (CLSI) [63, 64]. Minimum inhibitory concentrations were also determined using a low-salt Muller Hinton broth and the values were presented as MIC-ls. In brief, AvBDs were prepared in serial two-fold dilutions in a 96-well polypropylene microtiter plate and inoculated with the bacteria at a final concentration of 5 × 10^5^ CFU/ml. The final concentrations of AvBDs were ranging from 2 to 256 μg/ml. After incubation at 37 °C for 16 to 24 h, the lowest concentration that completely prevented visible bacteria growth was recorded. All antimicrobial assays were conducted in duplicate.

### Killing kinetics measurement

The killing kinetics of AvBD-6 and AvBD-12 were confirmed against *E. coli* ATCC 25922, *S.* Typhimurium ATCC 14028 and *S. aureus* ATCC 29213. Equal volumes (50 μl) of bacterial suspension at the concentrations 2 × 10^5^, 2 × 10^7^, and 2 × 10^9^ CFU/ml and AvBDs at a final concentration of 32 μg/ml were co-incubated in a 96-well polypropylene microtiter plate at 37 °C for 30, 60, 90, 120, 150, 180 min. Bacterial-peptide mixtures were serially diluted and plated on LB agar plates. Colonies were counted after incubation at 37 °C for 16 h. The assays were repeated three times with duplicate.

### Limulus amoebocyte lysate assay

The LPS-neutralizing abilities of AvBDs were assessed using a Limulus Amoebocyte Lysate (LAL) (Pierce™ LAL Chromogenic Endotoxin Quantitation Kit, Thermo Fisher Scientific) according to the manufacturer’s instructions. Briefly, equal volumes (25 μl) of AvBDs in endotoxin-free water (final concentrations of 8, 16, 32 μg) and *E. coli* O111:B4 LPS or *S.* Typhimurium L6143 LPS (2 EU/ml) were co-incubated at 37 °C for 30 min to permit binding of the peptide to LPS. Then, 50 μl of the assay mixture was transferred to Greiner CELLSTAR® flat-bottom, non-pyrogenic 96-well cell culture plate (VWR, Sugar Land, TX). Fifty microliters of 2-fold serially diluted *E. coli* O111:B4 LPS, ranging from 0.125 to 1.0 EU/ml, were used as the standards. Endotoxin-free water and AvBDs alone were included as negative controls. Fifty microliters of LAL was added to each well of the assay plate which was incubated at 37 °C for 10 min. One hundred μl of chromogenic substrate was then added to each well followed by incubation at 37 °C for 6 min, and addition of 50 μl of 25 % acetic acid to stop the reaction. Color change due to enzymatic liberation of *p*-Nitroaniline was monitored at 405 nm with Bio-Rad Benchmark Microplate Reader (Hercules, CA). LPS neutralizing rate was calculated as (OD405_AvBDs+LPS_ - OD405_H2O_)/(OD405_LPS_ - OD405_H2O_) × 100 %. The assays were performed three times with duplicate.

### Cell culture

Chicken macrophage MQ-NCSU and HD11 cell lines and Chinese hamster ovary (CHO)-K1 cell line were maintained in RPMI-1640 media supplemented with 10 % fetal bovine serum (FBS), 100 U/ml penicillin and 100 μg/ml streptomycin, at 37 °C in humidified air with 5 % CO_2_. Additional 2 % chicken serum was added for MQ-NCSU cells culture. Mouse immature dendritic cell line JAWSII (ATCC CRL-11904^TM^) was cultured in Alpha minimum essential medium with ribonucleosides, deoxyribonucleosides, 4 mM L-glutamine, 1 mM sodium pyruvate and 5 ng/ml murine Granulocyte macrophage colony-stimulating factor (GM-CSF), supplemented with 20 % FBS, 100 U/ml penicillin and 100 μg/ml streptomycin, at 37 °C in humidified air with 5 % CO_2_.

### Cell cytotoxicity

The cytotoxicity of AvBDs to four cell lines was determined using MTT (3-(4, 5-dimethylthiazol-2-yl)-2, 5-diphenyltetrazolium bromide) cell proliferation assay (Thermo Fisher Scientific). Cells (5 × 10^3^ cells/well) in 96-well microtiter tissue culture plates were treated with AvBD-6 and AvBD-12 at the concentrations of 4, 16, 64, and 256 μg/ml for 4, 12, 24, 48 h at 37 °C. After treatment, 20 μl of 12 mM MTT solution was added to each well and the palates were incubated for 4 h. The medium in each well was replaced with dimethyl sulfoxide (DMSO, Sigma-Aldrich) to dissolve MTT crystals. The plates were read using a spectrophotometer at 540 nm. The viability of treated cells was expressed as the percentage of viability relative to the untreated control. The experiment was performed in triplicate.

### Construction of pAcGFP1-N1-CCR2 and -CCR6

Total RNA was extracted from MQ-NCSU cells or chicken liver with the Rneay® Mini kit (Qiagen), and the first-stand cDNA was synthesized by reverse-transcription polymerase chain reaction (RT-PCR) with SuperScript® III first-strand synthesis system (Invitrogen) according the manufacturer’s instruction. PCR was performed using pfu DNA polymerase (Stratagene, CA) and primers containing flanking enzyme restriction sites *Xho*I and *Hind*III. The forward and reverse primers of CCR2 and CCR6 were as follows: CCR2-F 5´-CCGCTCGAGGCCACCATGGAGAACTATACTGACT-3´, CCR2-R 5´-CCCAAGCTTCAGTCCAGTAGAGATGTC-3´; CCR6-F 5´-CCGCTCGAGGCCACCATGAGTACTACAGTTTTTG-3´ and CCR6-R 5´-CCCAAGCTTTATAGTAAAAGAAGATGCAT-3´. The PCR products of CCR2 and CCR6 were purified and cloned to pCR® 2.1-TOPO® vector and transformed into *E. coli* TOP10F´ competent cells. Plasmid DNA was isolated from the transformed clones using the QIAprep® Spin Miniprep kit (Qiagen), and sequenced to confirm the correctness of inserts. The CCR2 and CCR6 inserts were subcloned to eukaryotic expression vector pAcGFP1-N1 (Clontech) between the *Xho*I and *Hind*III restriction sites and transformed into *E. coli* TOP10F´ competent cells. The recombinant pAcGFP1-N1-CCR2 and -CCR6 plasmids were extracted and confirmed by PCR and digestion with *Xho*I and *Hind*III.

### Transfection of CHO-K1 cells expressing CCR-2 and CCR-6

CHO-K1 cells were seeded into a 6-well-plate. After 80 % confluence, cells were transfected by pcGFP1-N1-CCR2, pAcGFP1-N1-CCR6 and pAcGFP1-N1 (mock) plasmids using the TransIT-CHO Transfection Kit according to the manufacturer’s instructions (Mirus Bio LLC, WI). After 72 h incubation, cells were harvested and selected in 500 μg/ml G8 (Sigma-Aldrich) media. CHO-K1 cells stably expressing CCR2 and CCR6 were used to perform chemotaxis assay.

To verify transfection, CCRs transfected CHO-K1 cells were cultured on a cover slide in RPMI-1640 with 10 % FBS, 2 mM L-glutamine, 100 units/ml penicillin and streptomycin, 500 μg/ml G8 at 37 °C in humidified air with 5 % CO_2_. After 80 % confluence, cells were washed with ice-cold phosphate buffered saline (PBS) and fixed by methanol:acetone (v:v, 1:1). Fixed cells were then washed twice with PBS and stained nuclei with 0.1 μg/ml DAPI (Thermo Fisher Scientific) in PBS for 1 min, then the cells were washed three times with PBS before microscopic visualization. Wild-type CHO-K1 cells and mock transfected cells were examined simultaneously as controls. Cells were visualized and captured using Nikon fluorescent microscope connected with Olympus DP2-BSE software (ECLIPSE E600, Japan, 20×). In addition, total RNAs were extracted from transfected cells and RT-PCRs were performed to verify expression of CCR2 and CCR6 as described in construction of pAcGFP1-N1-CCR2 and -CCR6. PCR products were subjected to gel electrophoresis and photographed on the FluorChem Q imaging system (Cell Biosciences, CA).

Western blot analysis was also carried out to confirm CCR2/CCR6 expression in CHO-K1 cells. In brief, CHO-K1 cells were suspended in RIPA cell lysis buffer (Thermo Fisher Scientific) for 10 min and centrifuged at 13,000 *g* for 10 min at 4 °C. Protein concentration was measured using a NanoDrop 1000 spectrophotometer at 280 nm (Thermo Fisher Scientific). An equivalent amount of protein from each sample was run on a 12 % SDS-PAGE gel and transferred onto a nitrocellulose membrane (Bio-Rad). The protein fractions were probed with polyclonal goat anti-GFP primary antibody (1:1000, Thermo Fisher Scientific) and horseradish peroxidase (HRP)-coupled anti-goat secondary antibody (1:500, Promega), and visualized after color development with 4-Chloro-1-Naphthol (4-CN) and 30 % hydrogen peroxide (Thermo Fisher Scientific). Protein bands were photographed using FluorChem Q imaging system (Cell Biosciences, CA).

### Chemotaxis assay

Migration of MQ-NCSU cells, JAWSII cells and CCR2-/CCR6-expressing CHO-K1 cells in response to chemotactic factors was determined using a 48-well microchemotaxis chamber technique as previously described [65]. Single cell suspension was prepared by treating cells with a non-enzymatic cell dissociation solution (Sigma-Aldrich), and the dissociated cells were harvested by centrifugation and resuspended in chemotaxis assay buffers at a final concentration of 1.5 × 10^6^ cells/ml. The chemotaxis buffer for MQ-NCSU and CCR2-/CCR6-expressing CHO-K1 cells was RPMI 1640 supplement with 0.1 % BSA, 100U/ml penicillin, and 100 μg/ml streptomycin, and for JAWSII cells was Alpha Minimum Essential Medium containing 0.1 % BSA, 100U/ml penicillin, and 100 μg/ml streptomycin. In brief, 28 μl of 4-fold diluted AvBDs in chemotactic buffer ranging from 0.25 to 64 μg/ml was placed in the lower wells of a 48-well microchemotaxis chamber (Neuro Probe, San Diego, CA), and 50 μl of cell suspension was added to the upper wells. The lower and upper compartments were separated by a pre-coated polycarbonate membrane (8 μm pore size) with 10 μg/ml fibronectin (BD biosciences, Bedford, MA). After incubation at 37 °C for 1 to 5 h in humidified air with 5 % CO_2_, the membranes were removed, topside scraped, stained with Kwik-Diff staining kit (Thermo Fisher Scientific), and counted under light microscopy. Chemotaxis buffer and bacterial peptide N-Formyl-methionyl-leucyl-phenylalanine (fMLF, Sigma-Aldrich) were used as negative and positive controls, respectively. The results were presented as chemotactic index (C.I.) using the following formula: C.I. = the number of migrated cells following treatment with AvBDs/the number of migrated cells following treatment with chemotactic buffer. To investigate if the peptides directly bind to plasma membrane components, JAWSII cells were pre-incubated with 0.25–64 μg/ml chemoattractant (AvBD-6 or AvBD-12) for 30 min at 37 °C in humidified air containing 5 % CO_2_. Then, the chemotaxis assay was performed with the same procedure.

For CCR2/6 expressing CHO-K1 cells, the mock transfected CHO-K1 cells were analyzed simultaneously as negative cell control. Recombinant chicken CCL20 (Innovative research, the chemokine ligand for CCR6) was used as positive chemotactic agent control. C.I. = the number of migrated CCR2/6-expressing CHO-K1 cells following treatment with AvBDs/the number of migrated cells following treatment with chemotactic buffer. All chemotaxis assays were performed five times.

### Reduction of AvBD peptides

Ten microgram of AvBDs were incubated with 0.8 mM β-nicotinamide adenine dinucleotide 2’-phosphate, reduced (NADPH), 0.2 μM thioredoxin reductase, 0, 1, 2, 4 μM *E.coli* thioredoxin (Sigma-Aldrich) at 37 °C for 60 min in 0.1 M potassium phosphate-2 mM EDTA buffer, pH 7.0. The incubation mixtures were analyzed using reversed-phase high performance liquid chromatography (RP-HPLC) and absorbance at 340 nm was monitored to test the consumption of NADPH using a NanoDrop 1000 spectrophotometer (Thermo Fisher Scientific). The reduced peptides were separated from thioredoxin system by Amicon Ultra centrifuge filters with 10 kDa membrane and 3 kDa membrane (Millipore). Natural and reduced AvBDs were analyzed by RP-HPLC using the Hitachi HPLC system and Phenomenex (Torrance, CA) Luna® C18 (2) Columns (100 × 4.6 mm, 3 μm), mobile phase 90 % A (water + 0.1 % (v/v) trifluoroacetic acid (TFA)) and 10 % B (acetonitrile + 0.1 % (v/v) TFA), under 214 nm UV wavelength, and a flow rate of 1.0 ml/min at 25 °C. The structures of AvBDs were probed by circular dichroism (CD) spectroscopy on a JASCO J-815 spectropolarimeter (JASCO, Easton, MD) in a 0.2 cm quartz cell. Spectra were recorded at peptide concentrations of 0.1 mg/ml diluted in a 10 mM phosphate buffer (pH 7.4), in far-ultraviolet (UV) region from 190 to 250 nm at room temperature. Each CD spectrum was the average of six consecutive scans, and all data were corrected with the blank buffer and expressed as molar ellipticity θ (deg · cm^2^ · mol^−1^).

### Transmission electron microscopy (TEM)

Mid-logarithmic phase *S.* Typhimurium cells (1 × 10^8^ CFU) were treated with wild-type and reduced AvBD-6 (16 μg/ml) and AvBD-12 (128 μg/ml) at 37 °C for 30 min in the presence of 5 mM NaCl. These AvBD concentrations were required to inhibit bacterial growth in the presence of 5 mM NaCl. Bacterial cells were harvested by centrifugation at 5, 000 *g* for 10 min. TEM imaging was performed according to the procedure described by Park and Kand [66]. In brief, samples were fixed in Karnovsky’s reagent (2 % paraformaldehyde, 2.5 % glutaraldehyde in 0.1 M sodium cacodylate buffer, pH 7.4) and dehydrated through a graded ethanol series of 20, 50, 70, 90 % and 3× 100 %, and then stained with 2 % uranyl acetate. Observations were made under a transmission electron microscope (JEOL 1400, Japan). Untreated cells were processed at the same time as negative control.

### Gel retardation assay

The binding of AvBDs to genomic DNA of *S.* Typhimurium was evaluated by gel retardation assay [37]. The genomic DNA of *S.* Typhimurium was extracted using Genomic DNA kit (Qiagen). AvBDs were mixed with 0.4 μg of genomic DNA at peptide-to-DNA ratios of 0, 0.5, 1, 2, 4 and 8 (w/w). BSA were used as negative control. The mixture were incubated for 10 min at room temperature and analyzed by electrophoresis on a 0.5 % agarose gel. Densitomeric analysis of photographed gel pictures was performed using ImageJ software (NIH, Bethesda, MD, USA. The data was expressed as the density ratio of migrated DNA with AvBDs or BSA binding to DNA without binding. The assay was performed in triplicate.

### Statistical analysis

Data were analyzed by student’s *t*-test or one-way analysis of variance (ANOVA) followed by Duncan’s test for multiple comparisons using software SPSS version 19.0 (IBM Corp., Armonk, NY), and expressed as means ± standard deviation (SD). Differences at *p* < 0.05 level were considered statistically significant, and at *p* < 0.01 level were considered extremely significant.

## Abbreviations

4-CN: 4-Chloro-1-Naphthol
AA: Amino acid
ANOVA: One-way analysis of variance
AvBD: Avian beta-defensin
BSA: Bovine serum albumin
C.I.: Chemotaxis index
CD: Circular dichroism
CFU: Colony-forming unit
DCs: Dendritic cells
FBS: Fetal bovine serum
fMLF: N-Formyl-methionyl-leucyl-phenylalanine
Fmoc: 9-fluorenyl-methoxycarbonyl
GFP: Green fluorescent protein
GM-CSF: Granulocyte macrophage colony-stimulating factor
HRP: Horseradish peroxidase
Ig: immunogluobin
LB: Luria-Bertani
LPS: Lipopolysaccharides
LTA: Lipoteichoic acid
MIC: Minimum inhibitory concentration
NADPH: Nicotinamide adenine dinucleotide phosphate
PBS: Phosphate buffered saline
RP-HPLC: Reversed-phase high performance liquid chromatography
SD: Standard deviation
TEM: Transmission electron microscopy
TLR4: Toll-like receptor 4
UV: Ultraviolet
